# Regulation of the Metal Transporters ZIP14 and ZnT10 by Manganese Intake in Mice

**DOI:** 10.3390/nu11092099

**Published:** 2019-09-04

**Authors:** Danielle M. Felber, Yuze Wu, Ningning Zhao

**Affiliations:** Department of Nutritional Sciences, The University of Arizona, Tucson, AZ 85721, USA (D.M.F.) (Y.W.)

**Keywords:** manganese, transporters, ZIP14, ZnT10, liver, intestine, homeostasis

## Abstract

The metal transporters ZIP14 and ZnT10 play key physiological roles in maintaining manganese (Mn) homeostasis. However, in vivo regulation of these two transporters by Mn is not understood. Here, we examined how dietary Mn intake regulates ZIP14 and ZnT10 by feeding mice a low-Mn diet, a control diet, or a high-Mn diet for 6 weeks. Inductively coupled plasma mass spectrometry was used to measure Mn and iron (Fe) levels. ZIP14 and ZnT10 protein levels were measured by western blot analysis. While mice on the high-Mn diet exhibited significantly higher levels of Mn in the blood, liver, and brain, the low-Mn diet group did not display matching reductions, indicating that high Mn intake is more effective in disrupting Mn homeostasis in mice. Additionally, Fe levels were only slightly altered, suggesting independent transport mechanisms for Mn and Fe. In the high-Mn diet group, ZIP14 and ZnT10 were both upregulated in the liver, as well as in the small intestine, indicating a coordinated role for these transporters in Mn excretion. Unexpectedly, this upregulation only occurred in male mice, with the exception of hepatic ZIP14, providing new insight into mechanisms behind widely observed sex differences in Mn homeostasis.

## 1. Introduction

Manganese (Mn) is an essential nutrient found at varying levels throughout the tissues and fluids in the body, with the highest concentrations found in the brain, liver, bone, pancreas, and kidney [1,2]. Mn functions as a cofactor for enzymes involved in many physiological processes, including gluconeogenesis, N-linked glycosylation, and urea formation [3]. It is also vital for the function of the mitochondrial antioxidant, Mn superoxide dismutase [4].

The recommended daily intake of Mn for adults is 2.3 mg/day for men and 1.8 mg/day for women [5]. Although only a small percentage of Mn is absorbed in humans, naturally-occurring Mn deficiency is uncommon as it is found in high levels in whole grains, legumes, rice, and nuts [6]. Mn homeostasis is believed to be largely maintained by hepatobiliary excretion, where absorbed Mn is taken up into the liver and conjugated to bile for excretion through the biliary duct and intestinal tract [7,8,9]. The mechanisms underlying Mn absorption remain unknown, but it is thought that regulation of whole-body Mn homeostasis occurs at least partially at the intestinal absorption level [10].

ZIP14 and ZnT10 are proteins that were first identified as transporters for zinc (Zn) and iron (Fe) [11,12]. However, in the past decade, studies have reported that individuals with loss-of-function mutations in these two transporters suffer from dysregulated Mn metabolism [13,14]. These findings suggest an important physiological role for these proteins in Mn homeostasis, but there is still limited understanding of the underlying mechanisms.

ZIP14 is highly expressed in the liver and small intestine in both mice and humans [15]. Those with loss-of-function mutations in *ZIP14* demonstrate hypermanganesemia along with progressive early-onset Parkinsonism-dystonia, indicating an indispensable role for ZIP14 in Mn homeostasis [14]. While Mn accumulates throughout the body in individuals with *ZIP14* mutations, the absence of Mn accumulation in the liver of these patients has guided a model where ZIP14 is responsible for transporting Mn into the liver for biliary excretion [14]. Similarly, *Zip14* knockout (KO) mice show Mn accumulation and coinciding motor deficits [16]. However, liver-specific *Zip14* KO mice did not exhibit this phenotype or severe Mn accumulation under normal dietary conditions, indicating that ZIP14 in other organs must play a physiological role in Mn metabolism [17]. In conjunction with this, ZIP14 localizes to the basolateral membrane of enterocytes [18,19]. Wildtype (WT) CaCo-2 cells demonstrate rapid basolateral-to-apical transport of Mn, but this transport was minimal in ZIP14-inactivated CaCo-2 cells [19]. Further, recently developed intestine-specific *Zip14* KO mice exhibited significant Mn accumulation in the liver and brain compared to WT mice, suggesting an important role for intestinal ZIP14 in regulating Mn absorption [19].

Similar to *ZIP14* mutations, mutations in *ZnT10* have been implicated in familial Mn-induced neurotoxicity leading to the onset of Parkinsonism [13,20]. ZnT10 is abundantly expressed in the liver, small intestine, and brain [12]. HeLa cells transfected with WT ZnT10 exhibited lower intracellular and higher extracellular Mn compared to controls, revealing ZnT10 as a Mn exporter [21]. While both *ZIP14* and *ZnT10* mutations result in hypermanganesemia, motor deficits, and neurodegeneration due to Mn accumulation in the brain, individuals affected by *ZnT10* mutations also present with high levels of hepatic Mn and liver cirrhosis [13,14]. These findings support a model where ZIP14 imports Mn into the liver and ZnT10 exports hepatic Mn into the bile canaliculi for biliary excretion.

However, recently it was shown that liver-specific *Znt10* KO mice only had a ~1.5-2.5-fold increase of Mn in the blood, liver, and brain compared to controls, while full-body *Znt10* KO mice exhibited a ~20-40-fold increase [22]. After detecting elevated expression of ZnT10 in the intestines, stomach, and esophagus of the liver-specific *Znt10* KO mice, it was hypothesized that ZnT10 in the gastrointestinal tract compensated for hepatic loss-of-function [22]. Supporting this idea, it was demonstrated that ZnT10 localizes to the apical domain of CaCo-2 cells, and ZnT10-overexpressing cells displayed increased apical Mn efflux [22]. Further, endoderm-specific *Znt10* KO mice displayed phenotypes and Mn levels comparable to that of the full-body KO mice [22]. Since the liver and the lining of the gastrointestinal tract are both derived from the endoderm [23], these new findings suggest that in addition to regulation of biliary excretion, ZnT10 in the intestine may play a role in regulating Mn homeostasis.

In the present study, we fed mice diets containing 0.1 ppm Mn, 20 ppm Mn, or 2000 ppm Mn to represent Mn deficiency, adequacy, and overload, respectively. These diets provided a model to analyze the impact of dietary Mn intake on Mn status and the regulation of Mn transporters, ZIP14 and ZnT10. We found that the high-Mn diet more drastically altered body Mn levels compared to the low-Mn diet. Mice on the high-Mn diet exhibited upregulation of both ZIP14 and ZnT10 in the liver, as well as in the small intestine. However, this upregulation was only clearly exhibited in male mice, with the exception of hepatic ZIP14. Our results provide new insights into the regulation of Mn metabolism.

## 2. Materials and Methods

### 2.1. Experimental Animals

Procedures for animal experiments were approved by the Institutional Animal Care and Use Committee. All mice were housed in the Laboratory Animal Facility at the University of Arizona. WT 129S6/SvEvTAC mice were weaned at 3 weeks of age, and for the following 3 weeks were fed a NIH-31 irradiated traditional rodent diet (Teklad 7913; Envigo, Indianapolis, IN, USA). At 6 weeks of age, the litters were divided by sex and assigned to one of three groups until each group contained 12 mice, with six males and six females. Each group was fed one of three AIN-93G purified animal diets modified to contain 0.1 ppm, 20 ppm, or 2000 ppm Mn (TD170653, TD160484, and TD170523; Envigo), representing a low-Mn diet, control diet, and high-Mn diet. Metal levels of each diet are presented in Figure S1. Cages contained 1–5 same-sex mice from the same litter, and were kept at 21–22 °C with 12-h light/dark cycles. Mice were provided tap water ad libitum and fed their respective diets for 6 weeks, with body weight and food consumption recorded every other week in the afternoon to control for time of day weight fluctuations. At 12 weeks of age, mice were euthanized by cardiac puncture under ketamine anesthesia for collection of whole blood and organs.

### 2.2. ICP-MS Metal Analysis

Inductively coupled plasma mass spectrometry (ICP-MS, Agilent 7700 ICP-MS, Santa Clara, CA, USA) was performed at the Arizona Laboratory for Emerging Contaminants to measure metal levels in diets, blood, and tissue from livers, spleens, and brains. Samples of each diet were digested in 3 mL concentrated nitric acid at room temperature overnight, followed by incubation at 85 °C for 4 h. Digested samples were then diluted to 3% nitric acid with Milli-Q water. 50 μL of each blood sample was digested in 1.95 mL 3% nitric acid and incubated at 85 °C for 4 h. Digested samples were centrifuged at 2000× *g* for 10 min and supernatant was collected.

Tissue samples were dried out through incubation at 85 °C for two days. Samples were then digested in 1 mL concentrated nitric acid at room temperature overnight, then at 60 °C for one day and finally at 80 °C until acid evaporated completely, with tubes vortexed daily. 300 μL concentrated nitric acid was diluted to 3% concentration with Milli-Q water for a final volume of 9 mL. Results were reported in ng/mL and multiplied by total volume, then divided by dry weight or μL to provide metal levels as ng/g dry tissue weight, ng/μL blood or μg/g diet sample weight.

### 2.3. Western Blot Analysis

Tissue samples were stored at ‒80 °C and homogenized in NETT buffer (150 mM NaCl + 5 mM EDTA + 10 mM Tris + 1% TritonX-100 in deionized H_2_O) with protease inhibitor. Small intestine samples were divided into six equal sections, and sections corresponding to the jejunum were used for lysis. Unfortunately, some small intestine samples degraded during lysis, so only undegraded samples were used for western blot analysis. Protein concentrations of the collected lysates were analyzed with the RC DC^TM^ Protein Assay (Bio-Rad Life Science, Hercules, CA, USA). 100–120 μg of protein from tissue lysates were separated by gel electrophoresis in a 10% SDS-polyacrylamide gel and electrophoretically transferred to nitrocellulose membranes (GVS, Sanford, ME, USA). Proteins were probed using our custom rabbit anti-mouse primary antibodies, a horseradish peroxidase (HRP)-conjugated anti-rabbit secondary antibody (NA9340; GE Healthcare, Chicago, IL, USA), and an HRP-conjugated GAPDH antibody for loading control (HRP-60004; Proteintech, Rosemont, IL, USA). Antibodies were diluted (ZIP14 1:2000, ZnT10 1:1000) in blocking buffer (5% Non-fat dried milk in TBST (Tris-buffered saline + Tween 20)). An enhanced chemiluminescent substrate (SuperSignal West Pico; Thermo Fisher Scientific, Waltham, MA, USA) was used for signal detection with the ChemiDoc™ MP Imaging System (Bio-Rad Life Science).

### 2.4. Generation of Antibodies

Since antibodies against mouse ZIP14 and ZnT10 are not commercially available, we utilized the method of expressing glutathione S-transferase (GST) fusion protein in *E. coli* to produce and purify the immunogens for the production of antibodies. Generation of the anti-mouse ZIP14 antibody has been described previously [19].

To construct a vector carrying the mouse ZnT10 (mZnT10) fusion protein, the sequence encoding C-terminal tyrosine 351 to phenylalanine 470 of mZnT10 protein was PCR amplified and cloned into pGEX-3X vector using BamHI and ECoRI restriction sites (forward primer, 5′-ATA ATG GAT CCA ATA CCA GGA TGC CAG CAG AAA AA-3′; reverse primer, 5′-AAT ATG AAT TCT CAA AAA TGA GTA CTG TTT TCA TAA TGT TGT CTC TC-3′).

GST-fusion proteins were expressed in *E. coli* and purified by affinity chromatography on gluthathione-Sepharose 4B (GE Healthcare). The immunization procedures were performed by the Pocono Rabbit Farm & Laboratory. The antisera obtained from the test bleeds were analyzed for mZIP14- and mZnT10-specific recognition by immunoblotting analyses. Antisera were cleared by glutathione Sepharose cross-linked with GST to bind and remove anti-GST antibody. The cleared flow-through fractions were collected and used to purify antigen-specific antibodies using glutathione Sepharose cross-linked with GST-fusion proteins. Both anti-mouse ZIP14 and anti-mouse ZnT10 antibodies were verified (Figure S2).

### 2.5. Statistical Analysis

Data was analyzed using PRISM 6 software (GraphPad, La Jolla, CA, USA). Equal variances were assessed using the Brown-Forsythe test. Differences in metal levels and protein levels among the three diet groups were analyzed using one-way analysis of variance (ANOVA) with Tukey’s post-hoc test. A *p*-value of <0.05 was considered statistically significant, with * *p* < 0.05, ** *p* < 0.01, and *** *p* < 0.001.

## 3. Results

### 3.1. Body Weights Do not Vary between Diet Groups

**Figure 1:**
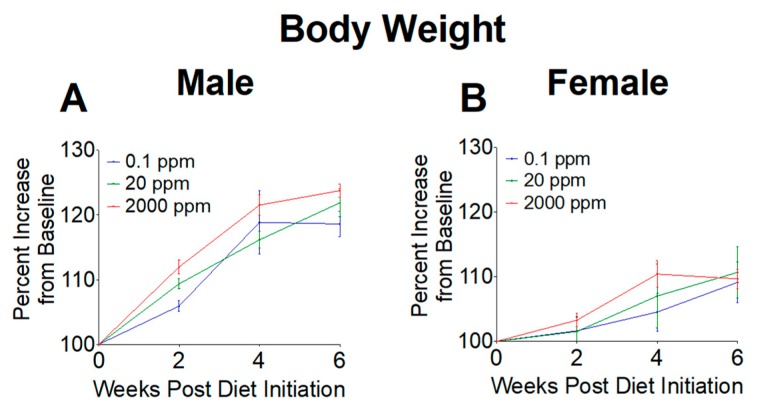
Relative body weights of mice upon initiation of 0.1, 20, and 2000 ppm Mn diets. (**A**) Male mice. (**B**) Female mice. Body weights expressed as percent increase from baseline during 6-week diet period (*n* = 6/sex). Data expressed as mean ± SEM.

Body weights were measured throughout our study to determine if high or low Mn intake altered weight gain. Weights were first measured at 6 weeks of age as baseline levels, then recorded every other week during the 6-week dietary intervention. Body weights were calculated as a percent increase from baseline. We found no significant differences in body weight gain throughout the 6-week intervention (Figure 1), indicating that mice grew at similar rates between the three diet groups.

### 3.2. Mn Levels are Elevated in Mice on the 2000 ppm Mn Diet

**Figure 2:**
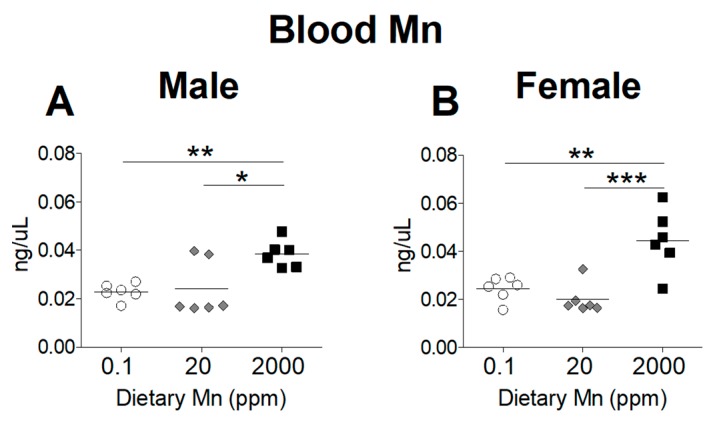
Whole blood Mn levels in mice on 0.1, 20, and 2000 ppm Mn diets. Mn content measured by inductively coupled plasma mass spectrometry (ICP-MS) in (**A**) male and (**B**) female mice at 12 weeks of age (*n* = 6/sex). Data expressed as mean ± SEM. * *p* < 0.05, ** *p* < 0.01, *** *p* < 0.001.

**Figure 3:**
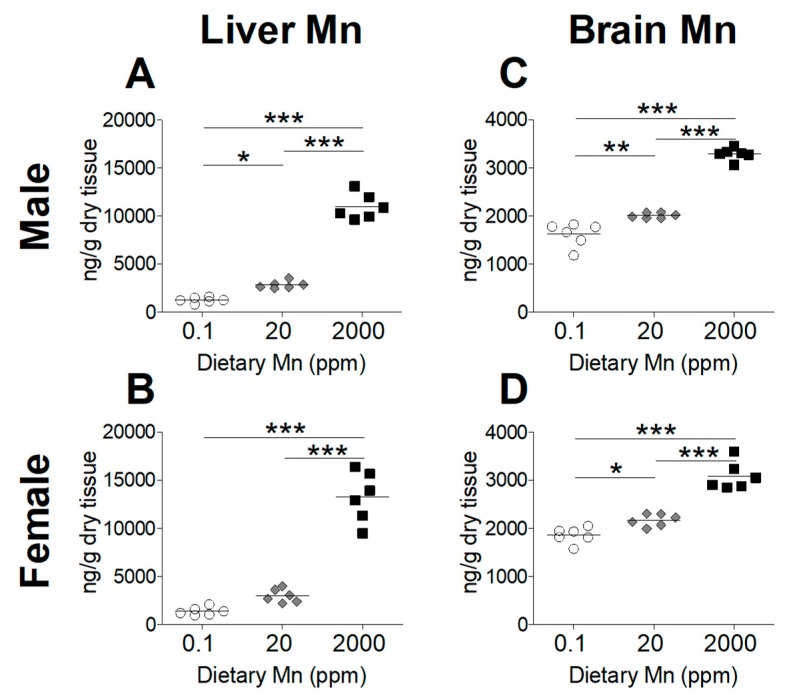
Tissue Mn levels in mice on 0.1, 20, and 2000 ppm Mn diets. Mn content measured by ICP-MS in male and female mice at 12 weeks of age (*n* = 6/sex). (**A** and **B**) Liver. (**C** and **D**) Brain. Data expressed as mean ± SEM. * *p* < 0.05, ** *p* < 0.01, *** *p* < 0.001.

To determine the effect of dietary Mn intake on body Mn status, we measured tissue Mn content by ICP-MS analysis, comparing mice on the high- and low-Mn diets (2000 ppm and 0.1 ppm) to those on the control diet (20 ppm). As expected, blood Mn levels were significantly increased in both sexes on the 2000 ppm Mn diet (Figure 2). However, a matching reduction of blood Mn was not observed for mice on the 0.1 ppm Mn diet (Figure 2). Liver Mn levels were significantly increased in the 2000 ppm Mn diet groups in both sexes (Figure 3A,B). The 0.1 ppm Mn diet decreased liver Mn content in male mice, but not in females (Figure 3A,B). Brain Mn levels in both sexes were significantly increased in the 2000 ppm Mn group, and were also slightly decreased in the 0.1 ppm Mn group (Figure 3C,D). These results show that high intake of Mn greatly increases body Mn loading, while low Mn intake for 6 weeks has less of an impact on body Mn status.

### 3.3. Fe Status Varies between Diet Groups

**Figure 4:**
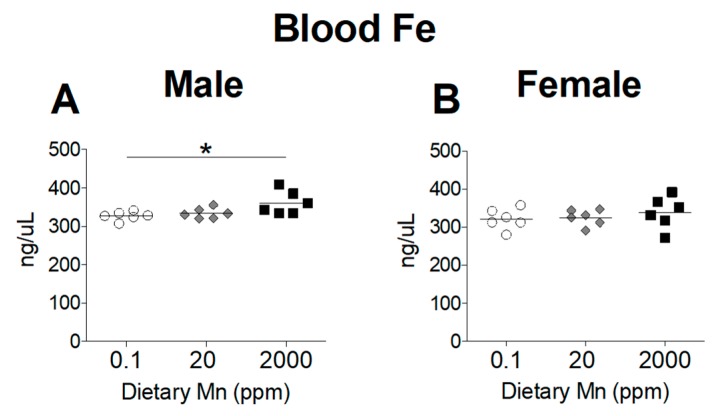
Whole Blood Fe levels in mice on 0.1, 20, and 2000 ppm Mn diets. Fe content measured by ICP-MS in (**A**) male and (**B**) female mice at 12 weeks of age (*n* = 6/sex). Data expressed as mean ± SEM. * *p* < 0.05.

**Figure 5:**
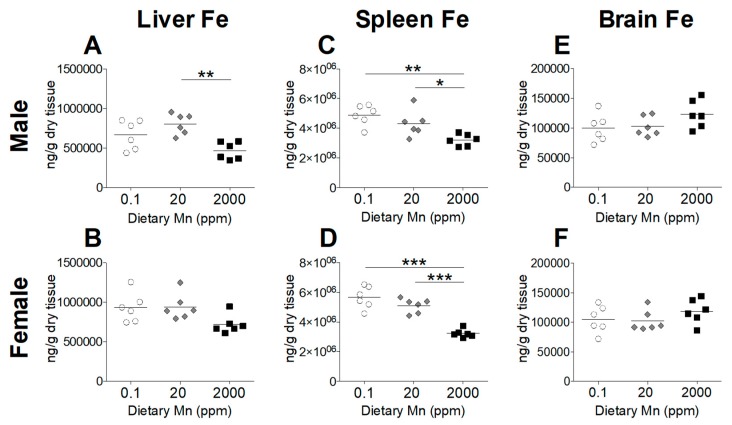
Tissue Fe levels in mice on 0.1, 20, and 2000 ppm Mn diets. Fe content measured by ICP-MS in male and female mice at 12 weeks of age (*n* = 6/sex). (**A** and **B**) Liver. (**C** and **D**) Spleen. (**E** and **F**) Brain. Data expressed as mean ± SEM. * *p* < 0.05, ** *p* < 0.01, *** *p* < 0.001.

Divalent metal transporter 1 (DMT1) is considered the primary transporter for Fe^2+^ absorption [24]. Competitive inhibition studies in K562 erythroleukemia cells show that Mn^2+^ and Fe^2+^ share the DMT1 pathway for cellular uptake and high concentrations of Mn reduces uptake of Fe^2+^ [24]. To determine if a high-Mn diet reduces body Fe levels and whether a low-Mn diet also influences Fe status, we measured Fe levels in the blood and other tissues. While varying Mn intake produced marked changes in Mn status, Fe status was only minimally affected. Blood levels of Fe were slightly increased in males on the 2000 ppm Mn diet, but only when compared to males on the 0.1 ppm Mn diet, with females having no statistically significant differences between the three groups (Figure 4). In the body, the liver and spleen are primary storage sites for Fe, and stored Fe becomes depleted before levels of circulating Fe decrease [25]. A moderate reduction in hepatic Fe was seen in males on the 2000 ppm Mn diet, while females did not show any statistically significant differences (Figure 5A,B). In the spleen, Fe levels were modestly reduced in males on the 2000 ppm Mn diet (−26%) and substantially reduced in females on the same diet (−37%) compared to those on the control diet (Figure 5C,D). No significant differences in Fe were observed in the brain (Figure 5E,F). The observed reductions of Fe in the liver and spleen of mice on the high-Mn diet suggest a release of storage Fe into the circulation, which may explain the lack of effect on blood Fe and the slight increase in males on the high-Mn diet compared to those on the low-Mn diet.

### 3.4. 2000 ppm Mn Diet Leads to Upregulation of Hepatic ZIP14

**Figure 6:**
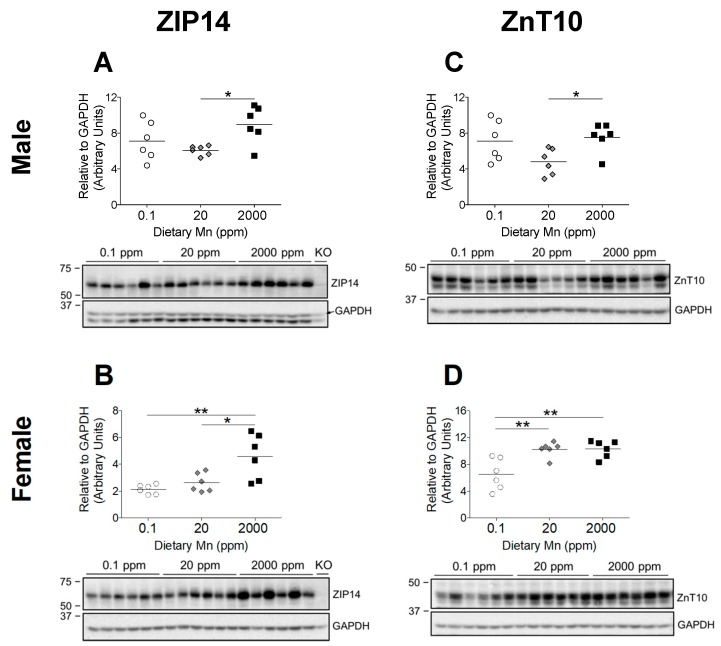
Comparison of transporter protein levels in the liver. Expression of (**A** and **B**) ZIP14 and (**C** and **D**) ZnT10 in male and female mice on 0.1, 20, and 2000 ppm Mn diets (*n* = 6/sex). Liver samples were obtained at 12 weeks of age and analyzed via western blot. Quantifications of protein levels were performed using GAPDH as a loading control. ZIP14 blots also include results from an age-matched *Zip14* knockout (KO) mouse as a control (fed traditional rodent diet). Data expressed as mean ± SEM. * *p* < 0.05, ** *p* < 0.01.

The significant increase in body Mn levels observed in the 2000 ppm Mn diet groups indicate that these mice were in a state of Mn overload, while the 0.1 ppm Mn diet groups displayed only slightly reduced body Mn (Figure 2 and Figure 3). Based on phenotypes of individuals with *ZIP14* and *ZnT10* mutations, it is clear that these transporters are required to maintain Mn homeostasis [13,14]. However, it is not known how these two transporters are regulated by Mn. Both ZIP14 and ZnT10 are highly expressed in the liver, which is a major organ involved in regulating Mn metabolism [12,15]. ZIP14 mainly localizes at the basolateral membrane of hepatocytes, while ZnT10 localizes to the canalicular domain of hepatocytes [26,27]. These two transporters work together to regulate hepatobiliary Mn excretion [27]. We aimed to establish whether a high-Mn diet would increase levels of ZIP14 and ZnT10 in the liver, and determine if they would similarly be downregulated by a low-Mn diet. We found that in both male and female mice, hepatic ZIP14 was significantly upregulated in the 2000 ppm Mn diet group compared to controls (20 ppm Mn) (Figure 6A,B). While males on the 0.1 ppm Mn diet showed varied hepatic ZIP14 levels, females on the 0.1 ppm Mn diet had more uniform ZIP14 levels, though neither were statistically significant (Figure 6A,B). These results suggest that ZIP14 functions to remove Mn from the body via biliary excretion when Mn is in excess.

### 3.5. Regulation of Hepatic ZnT10 Varies between Sexes

ZnT10 is believed to partner with ZIP14 to remove excess Mn via biliary excretion by exporting hepatic Mn into the bile [27]. Therefore, we hypothesized that hepatic ZnT10 in mice on the high-Mn diet will also increase. While male mice on the 2000 ppm Mn diet exhibited an upregulation of hepatic ZnT10 compared to the control diet group, ZnT10 levels were not significantly different in the 0.1 ppm Mn diet group (Figure 6C). Unexpectedly, females on the 2000 ppm Mn diet did not exhibit an upregulation of hepatic ZnT10, and instead the 0.1 ppm Mn diet group demonstrated a downregulation of ZnT10 (Figure 6D). The differences observed in hepatic ZnT10 regulation between male and female mice suggest that regulatory mechanisms for Mn excretion vary between sexes, which aligns with observations from human studies that females tend to have higher blood Mn levels than males [28,29,30].

### 3.6. Intestinal ZIP14 and ZnT10 are Upregulated in Males on 2000 ppm Mn Diet

**Figure 7:**
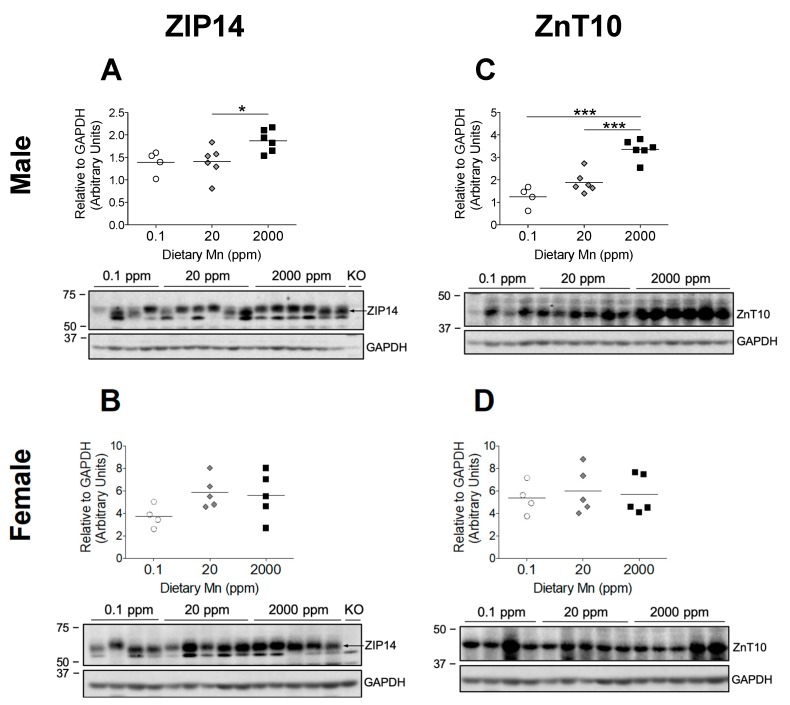
Comparison of transporter protein levels in the small intestine. Expression of (**A** and **B**) ZIP14 and (**C** and **D**) ZnT10 in male and female mice on 0.1, 20, and 2000 ppm Mn diets (*n* = 4–6/sex). Proximal sections of the small intestine were obtained during sacrifice at 12 weeks of age and samples that did not degrade during homogenization were analyzed via western blot. Protein quantifications were performed using GAPDH as a loading control. ZIP14 blots also include results from an age-matched *Zip14* knockout (KO) mouse as a control (fed traditional rodent diet). Data expressed as mean ± SEM. * *p* < 0.05, *** *p* < 0.001.

Hepatic ZIP14 and ZnT10 function together to prevent Mn accumulation in the body by promoting biliary Mn excretion, but liver-specific KO mice for both of these transporters do not display the same Mn accumulation seen in their full-body KO counterparts [17,22]. Recent cell studies indicate that ZIP14 promotes basolateral uptake of Mn in enterocytes, while ZnT10 promotes apical export of Mn, suggesting coordinated intestinal excretion of Mn by these two transporters [19,22]. Further, intestine-specific *Zip14* KO mice display significant Mn accumulation in the liver and brain, and endoderm-specific *Znt10* KO mice, which lack ZnT10 in both the liver and intestines, exhibit high Mn levels similar to that of full-body *Znt10* KO mice [19,22]. These findings suggest coordinated intestinal Mn excretion by these two transporters, but no studies have established the regulation of ZIP14 and ZnT10 in the intestine by dietary Mn. We found that male mice on the 2000 ppm Mn diet exhibited a significant upregulation of both ZIP14 and ZnT10 in the small intestine compared to the control diet group (Figure 7A,C), indicating that these transporters function together during Mn overload conditions to remove excess Mn by promoting intestinal basolateral-to-apical Mn secretion. Interestingly, like the results seen in hepatic ZnT10 regulation, females on the 2000 ppm Mn diet did not display upregulation of ZIP14 or ZnT10 in the small intestine (Figure 7B,D). There was also no difference in ZIP14 or ZnT10 levels for either sex on the 0.1 ppm Mn diet compared to mice on the 20 ppm Mn diet (Figure 7). These discrepancies between males and females suggest different regulatory mechanisms between the sexes which need to be further investigated.

## 4. Discussion

In the present study, we fed mice diets containing 0.1 ppm Mn (low-Mn), 20 ppm Mn (control), or 2000 ppm Mn (high-Mn), to determine if Mn intake influenced levels of Mn and Fe in a tissue-dependent manner, since coordinated regulation of these divalent metals has been suggested previously [24]. We also aimed to understand the roles for ZIP14 and ZnT10 in Mn homeostasis by revealing how these two transporters are regulated in response to Mn intake. Specifically, we examined the protein levels of these transporters in the liver, where they are known to regulate hepatobiliary Mn excretion, and in the small intestine, where they play roles secreting Mn into the lumen of the gastrointestinal tract [18,22].

To determine whether we had established an in vivo model for Mn deficiency or overload, we first measured Mn levels in the blood, liver, and brain. We observed that while mice on the diet containing 2000 ppm Mn showed significantly higher levels of Mn in the blood, liver, and brain, those on the 0.1 ppm Mn diet only exhibited a reduction of Mn in the brain, along with a minor reduction of Mn in the liver of male mice (Figure 2 and Figure 3). These findings indicate that mice are able to maintain adequate Mn levels even with a diet low in Mn for 6 weeks; however, excessive Mn acquired through increased dietary intake is not adequately removed and will accumulate throughout the body.

We also observed minor alterations in Fe levels along with increased intake of Mn. The potential mechanism could be related to DMT1. Although DMT1 is not required for Mn absorption [31], competitive inhibition studies in K562 erythroleukemia cells show that Mn^2+^ and Fe^2+^ share the DMT1 pathway for cellular uptake and high concentrations of Mn reduce uptake of Fe^2+^ [24]. This competitive inhibition has also been shown in humans. For example, a 7.5 mg Mn supplement reduced the absorption of a 3 mg Fe dose by 21%, while 15 mg Mn reduced Fe absorption by 34% [32]. Contrary to our expectations, we observed no differences in blood Fe in females, and a minor increase in blood Fe in males on the 2000 ppm Mn diet (Figure 4). In spite of the minimal effect in blood, we did see reduced Fe levels in the spleens of both sexes and livers of male mice on the 2000 ppm Mn diet (Figure 5A,C,D). Since hepatocytes, as well as splenic and hepatic macrophages are major storage sites of Fe, when Fe levels begin to decline, these cells mobilize stored Fe into the circulation to maintain adequate levels for metabolic requirements [25]. Therefore, despite normal Fe levels in the blood, the reduction of Fe seen in the liver and spleen from mice on the 2000 ppm Mn diet suggests that Fe deficiency could emerge following depletion of Fe from stores. However, these changes were still minor compared to changes seen in Mn levels, which is consistent with a recent report that DMT1 is not required for Mn absorption in mice [31].

Individuals with loss-of-function mutations in *ZIP14* display severe Mn overload, without Mn deposition in the liver [14]. The current model proposes that ZIP14 imports Mn into the liver to promote biliary excretion [14]. In our study, we observed hepatic Mn accumulation along with a significant increase of hepatic ZIP14 in mice on the 2000 ppm Mn diet (Figure 3 and Figure 6). These findings indicate that in response to diet-induced Mn overload, hepatic ZIP14 is upregulated and transports excess Mn into the liver for removal via biliary excretion.

Individuals with *ZnT10* mutations present with Mn overload along with increased hepatic Mn [13]. Given ZnT10’s function as a Mn exporter, it is believed that hepatic ZnT10 exports Mn into the bile canaliculi for excretion [21]. Our results suggest a sex difference in the regulation of hepatic ZnT10. We observed increased levels of ZnT10 in males on the 2000 ppm Mn diet compared to those on the control diet, yet no difference in ZnT10 levels for female mice on the high-Mn diet (Figure 6C,D). Surprisingly, the females with low Mn intake displayed decreased levels of hepatic ZnT10, while males in that group did not show significant differences compared to the control group (Figure 6C,D). The contrasting regulatory pattern of hepatic ZnT10 between sexes in mice on the high-Mn diet suggest that Mn metabolism differs between sexes, which will be further discussed below.

While ZIP14 and ZnT10 appear to be regulated by varying levels of Mn intake, their complementary roles promoting hepatobiliary excretion of Mn seem to be only part of their function. In the past few years, studies have revealed that both *Zip14* and *Znt10* liver-specific KO mice do not exhibit the same level of Mn accumulation as the full-body KO mice [17,22]. ZIP14 has been shown to localize to the basolateral membrane of mouse intestines and CaCo-2 cells, and functions to transport Mn from the basolateral to the apical side of enterocytes [18,19]. ZnT10 localizes to the apical domain of CaCo-2 cells, and functions as a Mn exporter to secrete Mn into the lumen [22]. Further, intestine-specific *Zip14* KO mice show elevated Mn in the liver and brain, and endoderm-specific *Znt10* KO mice, which lack ZnT10 in both the liver and intestines, exhibit Mn accumulation comparable to that of full-body *Znt10* KO mice [19,22]. These important findings suggest that both intestinal ZIP14 and ZnT10 facilitate Mn secretion or limit Mn absorption, with ZIP14 transporting blood Mn into the enterocyte and ZnT10 transporting Mn from the enterocyte into the intestinal lumen. We aimed to characterize the regulation of these transporters in the intestine by dietary Mn to elucidate how Mn homeostasis might be controlled via intestinal absorption. We found that in response to high Mn intake, both ZIP14 and ZnT10 are significantly upregulated in the small intestine of male mice, indicating that these transporters likely facilitate intestinal Mn excretion under high Mn conditions (Figure 7). Interestingly, female mice on the high-Mn diet did not show this same upregulation of intestinal ZIP14 and ZnT10, displaying levels similar to those seen in mice on the control diet (Figure 7). These findings may provide one potential mechanism for the sex differences in Mn absorption observed in earlier human studies [6]. While our results reveal altered ZIP14 and ZnT10 protein levels, it would be beneficial for future studies to measure protein levels exclusively in the plasma membrane to determine if the transporter proteins also correctly localized to the cell membrane.

Sex differences in Mn metabolism are well-documented [6,28,29,30]. Following a Mn-containing meal, men absorbed 1.35 ± 0.51% of ingested Mn, while women absorbed 3.55 ± 2.11% [6]. Accordingly, it has also been widely reported that women tend to have higher blood Mn levels than men [28,29,30]. Differences in Mn-regulating transporters may explain this disparity. In a study of *Znt10* KO mice, male KO mice were noted to have greater hepatic Mn levels than female KO mice, and male KO mice weighed 60% less than their corresponding WT controls, while females only weighed 40% less [33]. Observations also suggested that male KO mice died earlier than the females [33]. Likewise, in a study of *Zip14* KO mice, ZIP14 loss-of-function led to a greater accumulation of Mn in males than in females [16]. The researchers also reported that following an oral gavage of ^54^Mn, intestinal levels of ^54^Mn in female KO mice did not differ from the WT controls; however, levels in the WT males were significantly greater than in the KO males [16]. In our study, Mn-overloaded males exhibited an upregulation of ZIP14 and ZnT10 in both the liver and small intestine, while the females only showed an upregulation of ZIP14 in the liver and no upregulation of ZnT10 in either organ (Figure 6 and Figure 7). This higher activity of ZIP14 and ZnT10 in male mice supports the observations that male *Zip14* KO and *Znt10* KO mice are more severely impacted and exhibit greater Mn accumulation than females. Taken together, these findings indicate that ZIP14 and ZnT10 may play a larger role in regulating Mn homeostasis in males than in females during high Mn conditions. These results shed new light on the potential mechanisms underlying sex differences in Mn metabolism and homeostasis.

## 5. Conclusions

**Figure 8:**
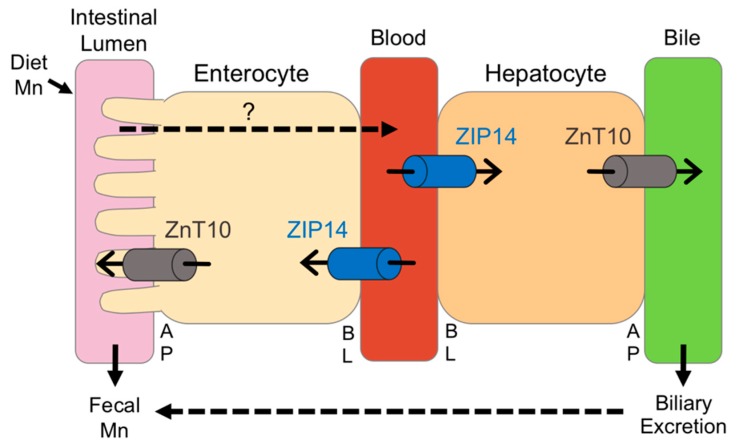
Proposed model for the regulation of ZIP14 and ZnT10 under high-Mn conditions. The transporters required for dietary Mn absorption across the apical (AP) and basolateral (BL) membranes of enterocytes are not fully established. In the intestine, ZIP14 transports blood Mn into the enterocyte, while ZnT10 secretes Mn from the enterocyte into the intestinal lumen, both limiting absorption. In the liver, ZIP14 takes up Mn from the blood into the hepatocyte, while ZnT10 secretes Mn into the bile. In male mice, when dietary intake of Mn is high, intestinal ZIP14 and ZnT10 are upregulated to reduce absorption, and hepatic ZIP14 and ZnT10 are upregulated to increase biliary excretion. In female mice, however, only hepatic ZIP14 is significantly upregulated during high dietary Mn conditions.

In summary, we utilized high and low dietary intake of Mn to examine the relationship between Mn and Fe, and investigate the regulation of ZIP14 and ZnT10. While these diets significantly altered body Mn levels, with the high-Mn diet having a much stronger impact, we only observed minor changes in Fe levels, indicating that the regulation of these metals may not be as tightly coupled as formerly believed. Most previous studies have been limited to studying in vivo regulation of ZIP14 and ZnT10 at the transcript level, but our custom antibodies against these transporters allowed us to measure their protein levels. To our knowledge, this study is the first to establish in vivo regulation of ZIP14 and ZnT10 by Mn. We demonstrated that in response to a high-Mn diet, ZIP14 and ZnT10 are upregulated in the liver, confirming their role in hepatobiliary excretion of Mn. The increase in intestinal ZIP14 and ZnT10 seen in response to the high-Mn diet substantiates previous proposals that these transporters regulate intestinal Mn excretion to prevent overload. However, lack of upregulation of these transporters in female mice provides new insight into the underlying mechanisms behind sex differences in Mn metabolism. We propose that ZIP14 and ZnT10 in the liver and small intestine work cooperatively to manage excretion of excess Mn, but this may occur to a greater extent in males given the significant upregulation of these transporters in males on the high-Mn diet, which was not shown in females, with the exception of hepatic ZIP14 (Figure 8). This may explain why previous studies have shown higher absorption rates as well as higher blood levels of Mn in females. These results broaden current knowledge of Mn metabolism and provide potential explanations for widely observed sex differences in Mn homeostasis.

## Supplementary Materials

The following are available online at https://www.mdpi.com/2072-6643/11/9/2099/s1, Figure S1: Metal Levels in Animal Diets. Metal levels measured by inductively coupled plasma mass spectrometry (ICP-MS) in AIN-93G purified animal diets modified to contain 0.1 ppm, 20 ppm, or 2000 ppm Mn. (**A**) Mn levels. (**B**) Fe levels. (**C**) Zn levels. (**D**) Cu levels. Data expressed as mean ± SEM, Figure S2: Antibody Verification. (**A**) To confirm the specificity of our mZIP14 antibody, we measured ZIP14 expression in the livers of a wildtype (WT) mouse, a Zip14 knockout (KO) mouse, and two heterozygous (HZ) mice, using GAPDH as a loading control. We demonstrated strong signal for the ZIP14 protein in the WT mouse, no signal in the KO mouse, and partial signal in the HZ mice, verifying our anti-mZIP14 antibody. (**B**) As we do not currently have *Znt10* KO mice, we confirmed the specificity of this antibody by transfecting HEK293 cells with an empty vector (CON), and a vector encoding mouse ZnT10 (mZnT10) with a FLAG epitope (mZnT10-FLAG). Cells were lysed 48 h after transfection and lysates were analyzed by immunoblotting (IB) with anti-FLAG or anti-mZnT10 antibodies. Both β-ACTIN and GAPDH were used as loading controls., Figure S3: Uncropped Western Blot Images for Figure S2, Figure S4: Uncropped Western Blot Images for Figure 6, Figure S5: Uncropped Western Blot Images for Figure 7.
