# Supplementary material for: Regulation of the Metal Transporters ZIP14 and ZnT10 by Manganese Intake in Mice
Source: Nutrients. 2019 Sep 4;11(9):2099. doi: 10.3390/nu11092099 (PMC6770778; doi:10.3390/nu11092099)
Supplement: Supplementary file 1 [file nutrients-11-02099-s001.pdf]

nutrients

Supplementary Materials

Regulation of the Metal Transporters ZIP14 and ZnT10 by Manganese Intake in Mice

Danielle M. Felber, Yuze Wu and Ningning Zhao *

Department of Nutritional Sciences, The University of Arizona, Tucson, AZ 85721; dmfelber@gmail.com; yuzewu@email.arizona.edu; zhaonn@email.arizona.edu

* Correspondence: zhaonn@email.arizona.edu; Tel.: +1-520-621-9744

List of materials included: Figures S1–S5

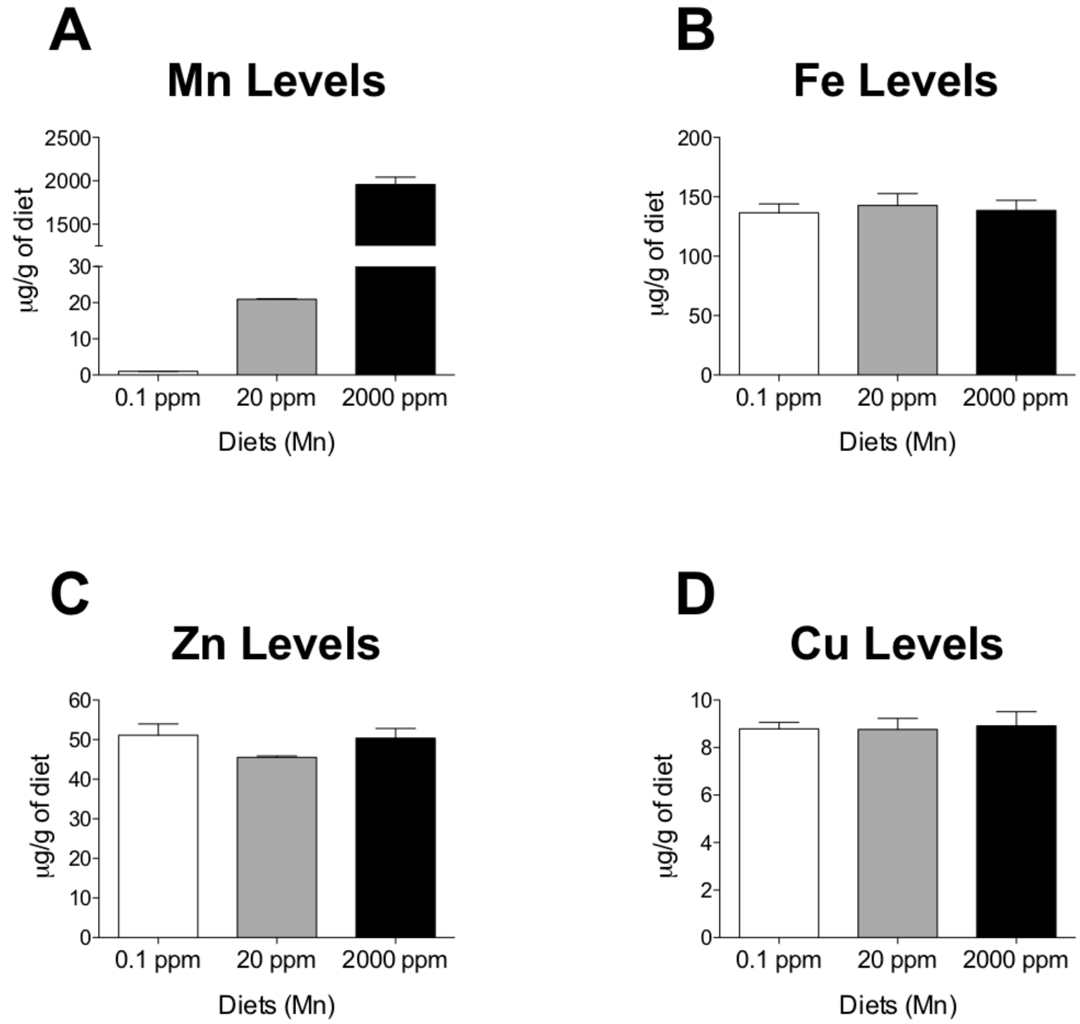

Figure S1. Metal Levels in Animal Diets. Metal levels measured by inductively coupled plasma mass spectrometry (ICP-MS) in AIN-93G purified animal diets modified to contain 0.1 ppm, 20 ppm, or 2000 ppm Mn. **(A)** Mn levels. **(B)** Fe levels. **(C)** Zn levels. **(D)** Cu levels. Data expressed as mean ± SEM.

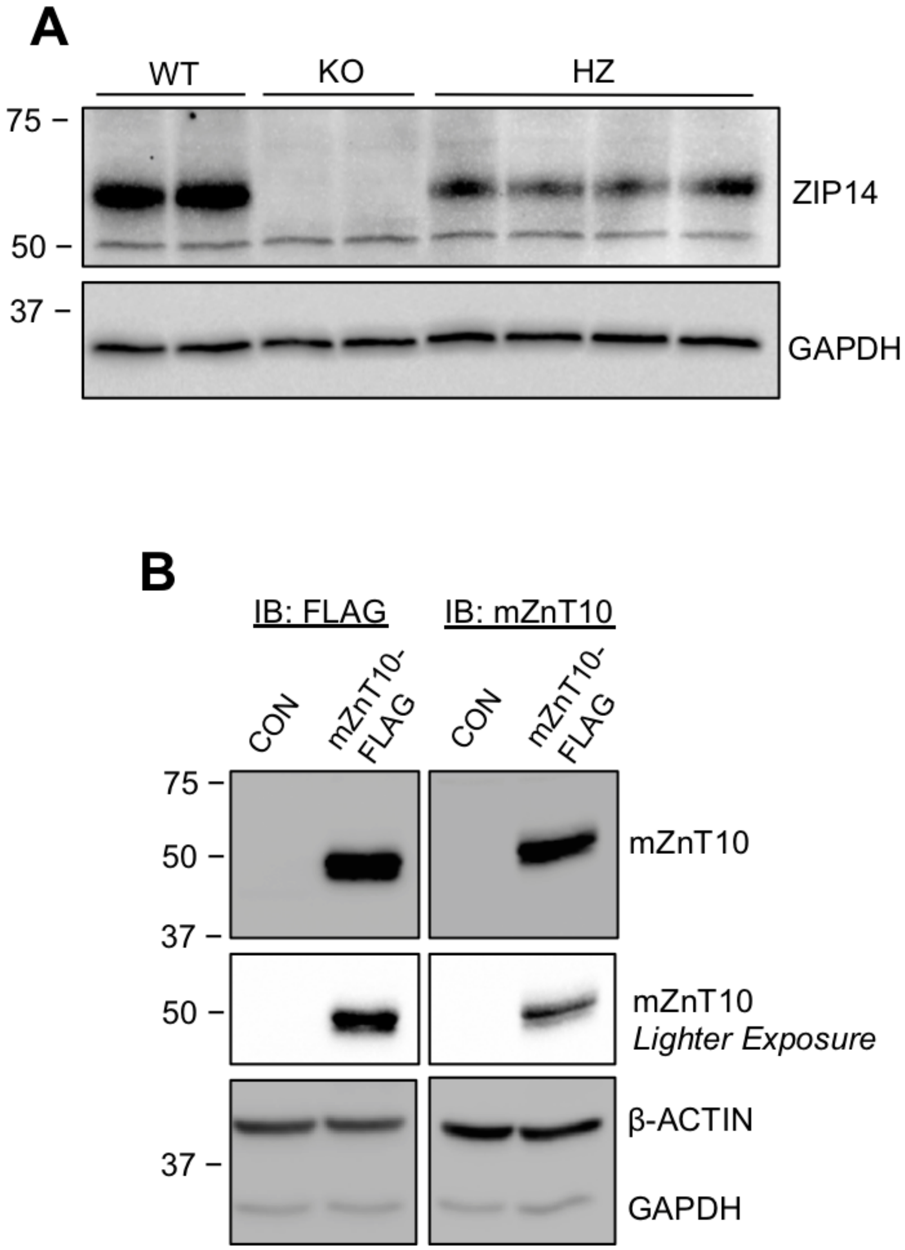

Figure S2. Antibody Verification. (A) To confirm the specificity of our mZIP14 antibody, we measured ZIP14 expression in the livers of a wildtype (WT) mouse, a *Zip14* knockout (KO) mouse, and two heterozygous (HZ) mice, using GAPDH as a loading control. We demonstrated strong signal for the ZIP14 protein in the WT mouse, no signal in the KO mouse, and partial signal in the HZ mice, verifying our anti-mZIP14 antibody. **(B)** As we do not currently have *Znt10* KO mice, we confirmed the specificity of this antibody by transfecting HEK293 cells with an empty vector (CON), and a vector encoding mouse ZnT10 (mZnT10) with a FLAG epitope (mZnT10-FLAG). Cells were lysed 48 hours after transfection and lysates were analyzed by immunoblotting (IB) with anti-FLAG or anti-mZnT10 antibodies. Both β-ACTIN and GAPDH were used as loading controls.

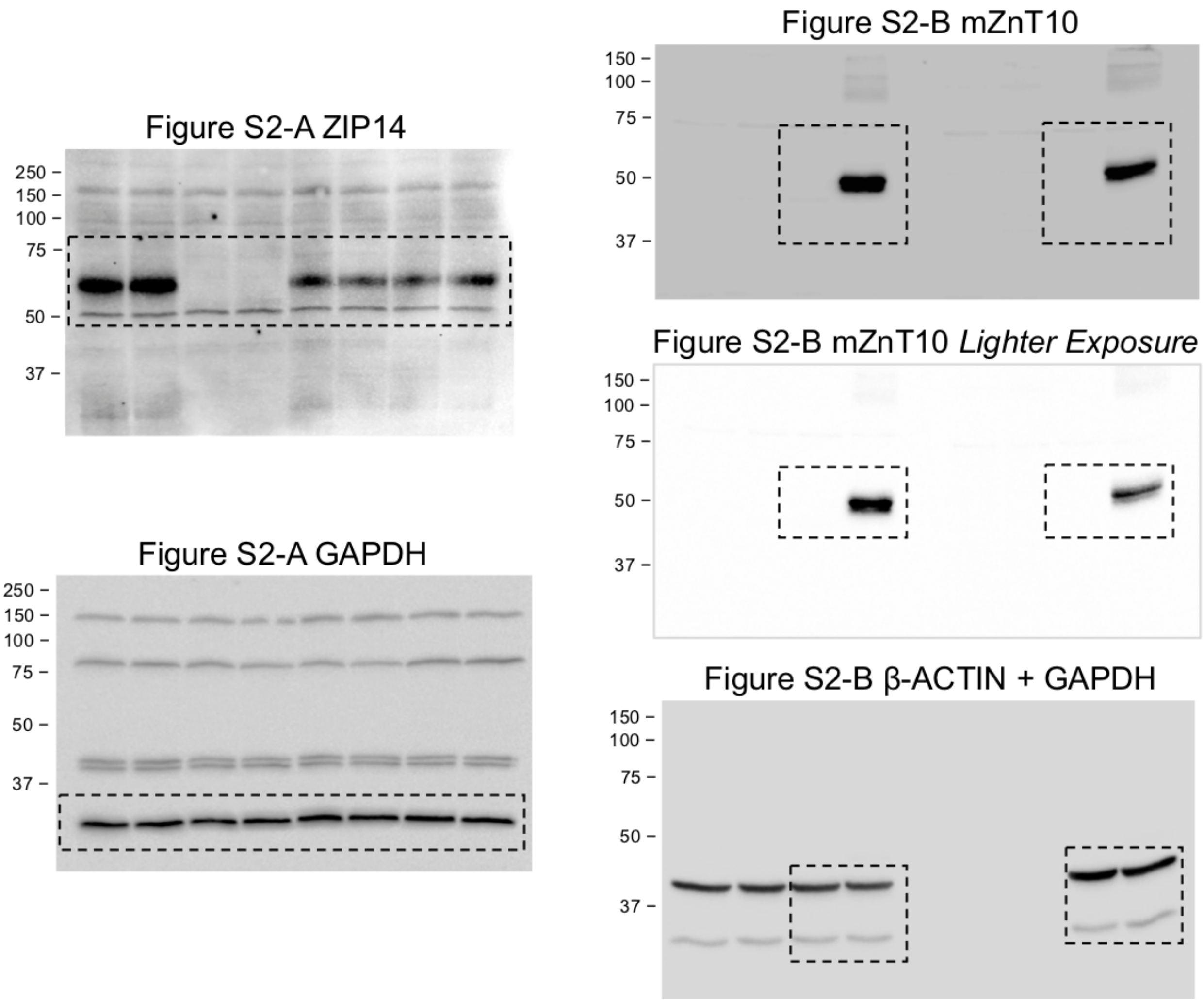

Figure S3. Uncropped Western Blot Images for Figure S2.

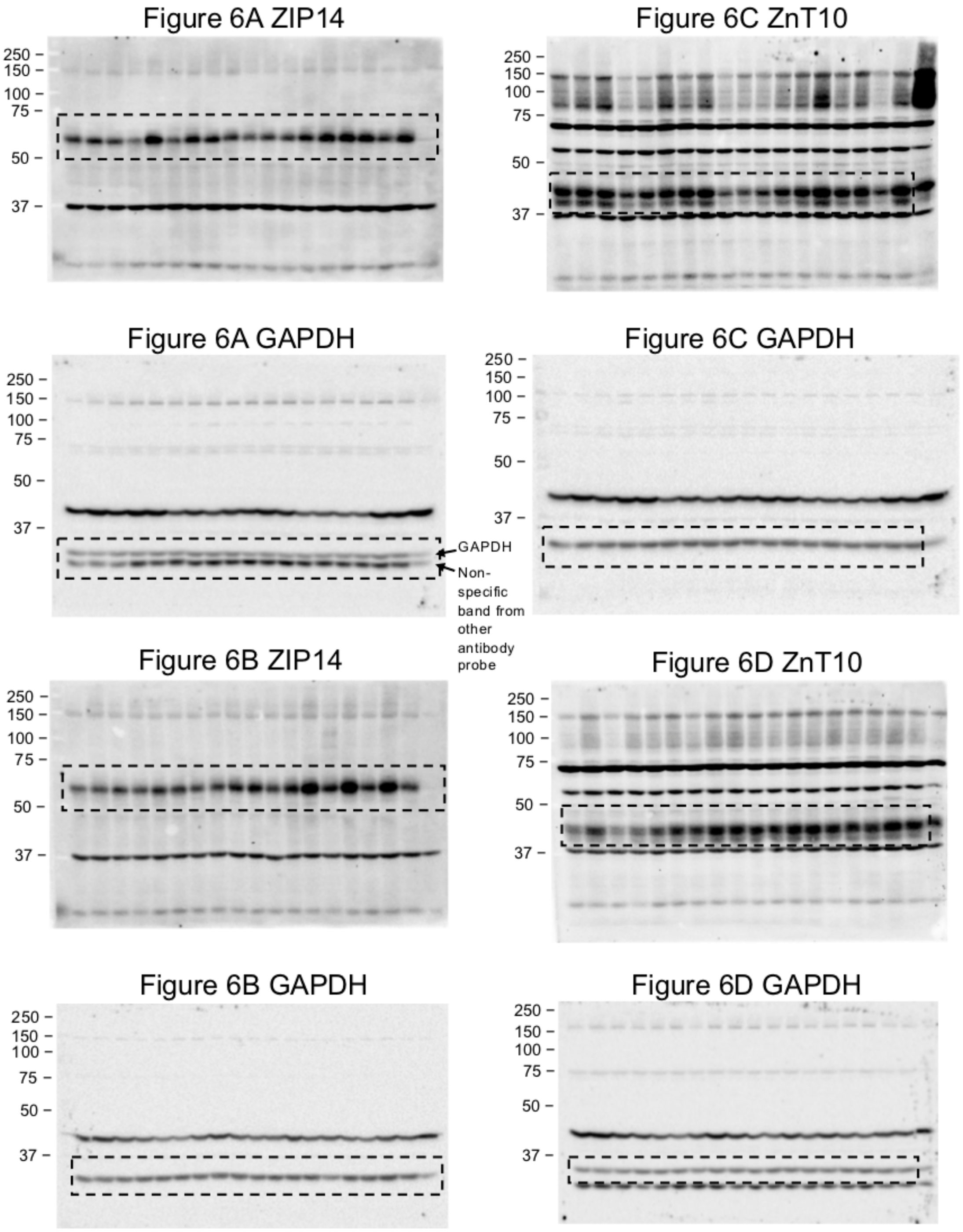

Figure S4. Uncropped Western Blot Images for Figure 6.

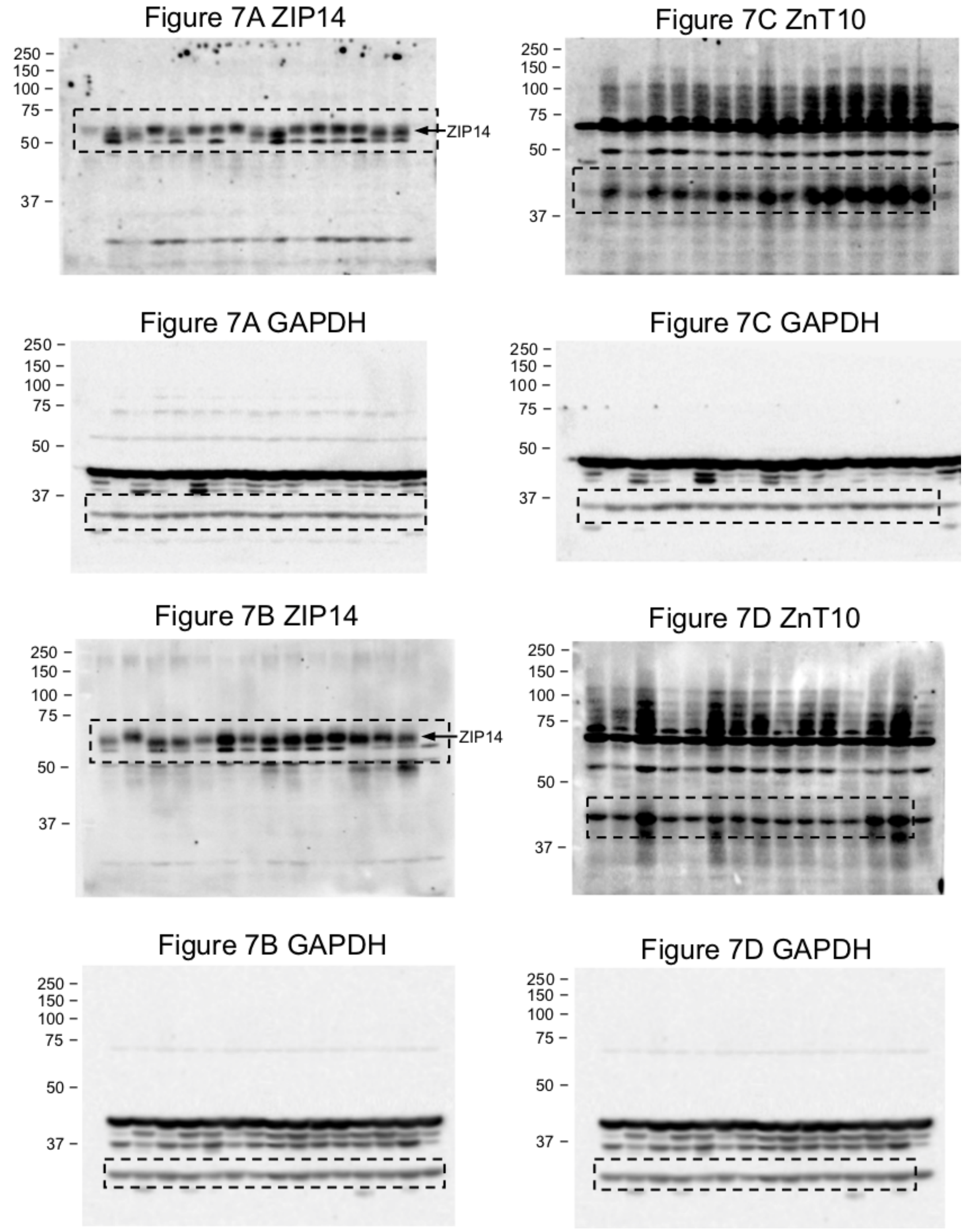

Figure S5. Uncropped Western Blot Images for Figure 7.
